# Analysis of educational research at a medical faculty in Germany and suggestions for strategic development – a case study

**DOI:** 10.3205/zma001070

**Published:** 2016-11-15

**Authors:** Sarah Prediger, Sigrid Harendza

**Affiliations:** 1 University Hospital Hamburg-Eppendorf, III. Medical Clinic, Hamburg, Germany

**Keywords:** educational research, medical education, research networks, research projects, strategic development

## Abstract

**Background:** Evidence-based medical education is playing an increasingly important role in the choice of didactic methods and the development of medical curricula and assessments. In Germany, a growing number of educational research projects has accompanied an ongoing change in the medical education process. The aim of this project was to assess medical education research activities at one medical faculty to develop procedural recommendations for the support and development of best evidence medical education.

**Methods:** Using a newly developed online questionnaire, the 65 institutes and departments of the medical faculty of Hamburg University at Hamburg University Medical-Center (UKE) were asked to report their medical education research and service projects, medical education publications, medical education theses, financial support for educational projects, and supportive structures that they would consider helpful in the future. The data were grouped, and a SWOT analysis was performed.

**Results: **In total, 60 scientists who were involved in 112 medical education research publications between 1998 and 2014 were identified at the UKE. Twenty-five of them had published at least one manuscript as first or last author. Thirty-three UKE institutions were involved in educational service or research projects at the time of the study, and 75.8% of them received internal or external funding. Regular educational research meetings and the acquisition of co-operation partners were mentioned most frequently as beneficial supportive structures for the future.

**Conclusion: **An analysis to define the status quo of medical education research at a medical faculty seems to be a helpful first step for the development of a strategy and structure to further support researchers in medical education.

## Zusammenfassung

**Hintergrund:** Evidenzbasierte medizinische Ausbildung spielt eine immer wichtiger werdende Rolle bei der Auswahl didaktischer Methoden und der Entwicklung medizinscher Curricula und Prüfungen.

In Deutschland hat eine wachsende Zahl von Ausbildungsforschungsprojekten den fortschreitenden Wandel des medizinischen Ausbildungsprozesses begleitet. Ziel dieses Projektes war es, die medizinischen Ausbildungsforschungsaktivitäten an einer medizinischen Fakultät einzuschätzen, um Prozessempfehlungen zur Unterstützung und Entwicklung von evidenzbasierter medizinischer Ausbildung zu entwickeln.

**Methoden: **Mit einem neu entwickelten Online-Fragebogen wurden 65 Institute und Abteilungen der Medizinischen Fakultät der Universität Hamburg am Universitätsklinikum Hamburg-Eppendorf (UKE) gebeten, über ihre Ausbildungsforschungs- und Serviceprojekte, ihre Publikationen in der Ausbildungsforschung, Promotionsarbeiten im Bereich der Ausbildungsforschung, Fördermittel für Ausbildungsprojekte und unterstützende Strukturen, die sie als zukünftig hilfreich einstufen würden, zu berichten. Die Daten wurden zusammengestellt und es wurde eine SWOT-Analyse durchgeführt.

**Ergebnissen: **Insgesamt 60 Wissenschaftler_innen des UKE waren an 112 Publikationen im Bereich der medizinischen Ausbildungsforschung zwischen 1998 und 2014 beteiligt. 25 von ihnen haben mindestens ein Manuskript in Erst- oder Letztautorenschaft veröffentlicht. 33 der Abteilungen am UKE waren an ausbildungsbezogenen Service- oder Ausbildungsforschungsprojekten beteiligt und 75.8% von ihnen erhielten interne oder externe Fördermittel. Regelmäßige Treffen zu Ausbildungsforschungsthemen und Unterstützung bei der Suche nach Kooperationspartner_innen wurden am häufigsten als nützliche unterstützende Strukturen für die Zukunft genannt.

**Schlussfolgerung: **Eine Erhebung zur Feststellung des Ist-Zustandes der medizinischen Ausbildungsforschung an einer medizinischen Fakultät scheint ein hilfreicher erster Schritt zur Entwicklung von Strategie und Struktur zur weiteren Unterstützung von Forscher_innen in der medizinischen Ausbildungsforschung zu sein.

## Introduction

Since a major revision of the medical licensure regulation in 2002 [Approbationsordnung für Ärzte (ÄAppO), accessed: 05.11.2015], medical education in Germany began to develop in an irreversible process [1], which is still ongoing today. The most common changes in educational and assessment structures at many medical faculties have been the implementation of problem-orientated learning (POL), objective structured clinical examinations (OSCE), and communication training with standardized patients [2], [3], [4]. Furthermore, medical faculties or individual departments of medical faculties have established studies to investigate whether changes in their curricula have improved undergraduate medical training [5], [6]. In addition, many medical faculties have initiated didactic teacher training programs [7], [8], [9], and a master of medical education program has been established in Germany to professionalize medical educators [10].

The German Council of Science and Humanities released recommendations for the further development of medical education in Germany based on an inventory of model curricula for undergraduate medical education [11]. These recommendations, which focus especially on the development of curricula in which basic and clinical sciences are integrated with a special focus on practical skills and communication, are greatly appreciated, but a more evidence-based approach has been demanded [Pfeilschifter J. Wissenschaft in hoher Verdünnung, accessed: 07.04.2016]. Furthermore, the Medical Faculty Association (MFT) has recently passed a National Catalogue of Competency Based Learning Objectives (NKLM) for medical schools [NKLM, accessed: 17.07.2015] and dental schools [NKLZ, accessed: 07.04.2016], which will foster a new approach to undergraduate medical education based on competences and entrustable professional activities [12], and it might trigger further curricular reform.

Research in medical education supports evidence-based medical education and facilitates the development of medical curricula based on the best evidence-based standards [13], [14]. As the impetus for curricular development, evidence-based medical education has a long tradition in North America and some European countries like Great Britain and the Netherlands, where departments of educational research and development are well established at medical faculties [15]. Hence, these countries are among those with the highest relative productivity with respect to scientific publications in educational research [16]. German universities are starting to establish Departments of Educational Development and Research in their Medical Faculties, e.g., in Munich. Additionally, with the implementation of the NKLM on trial for five years, educational research activities are needed for its assessment in practice. Furthermore, the NKLM itself emphasizes the scientific nature of medical education. In addition, the German GMS Journal for Medical Education, the scientific journal of the Society for Medical Education (GMA) to promote evidence-based medical education, celebrated its 30^th^ anniversary in 2014 [17]. In addition, since 2010, the “Ars legend-Faculty Award for excellent teaching in Medicine” is given to outstanding medical teachers and researchers in medical education by the Association for the Promotion of Science and Humanities in Germany and the MFT [18]. 

Despite such positive developments and support for educational research in medicine, and in contrast to expectations, the number of contributions to AMEE (Association of Medical Education in Europe) conferences by participants from German-speaking countries has not shown a continuous increase between 2005 and 2013 [19]. The number of publications by German education researchers in international medical education journals has increased since 2009 [20]. However, between 2004 and 2013, only five German universities published more than ten manuscripts with a German first or last author in an international medical education journal [20].

Meanwhile, at least one faculty member of every medical faculty in Germany completed a Master of Medical Education program [10], and many more have attended workshops for education research in medicine [21]. To increase the international visibility of medical educational research, establish evidence-based curriculum development activities and promote educational research at medical faculties with a network of educational researchers, it might be a useful approach to investigate the “status quo” of educational research at a medical faculty and to develop strategic steps from such an analysis to bundle education research activities. Our study describes the process used to analyze medical education research and development activities at the Medical Faculty of Hamburg University and the development of strategic ideas based on this analysis to promote evidence-based medical education at this faculty.

## Methods

The planning of a strategic development process is usually based on a thorough analysis of the specific situation of an organization preparing for change to customize recommendations for the next steps with the most precision [22], [23]. Therefore, we developed the following strategy for the analysis of medical education research and development activities at the Medical Faculty of Hamburg University including an online questionnaire, telephone interviews, and an internet based search. We designed an online questionnaire consisting of 23 mostly closed-ended questions with the option to include additional aspects in open questions. The questions included the following topics:

1. currently active education researchers per institution,
2. publications in educational research (journals with or without impact factors, books),
3. current education projects (research or development, topics, methods, target groups),
4. funding for education projects (funding source, amount),
5. promotion of young educational researchers (PhD program, MD thesis),
6. current partners for cooperation
7. expectations of an organizational structure for medical education at a medical faculty.

Furthermore, open questions were asked with respect to additional needs and suggestions regarding the possible establishment of a network for medical education at the University Hospital Hamburg-Eppendorf (UKE). Additional information regarding research activities, funding or memberships was collected from the dean of education’s secretary and from the UKE website (see attachment 1 ).

A link to the online questionnaire was sent by email to the teaching coordinator of every department (n=37) and institute (n=27) at the UKE and to the executive director of the vice deanship of education of the Medical Faculty of Hamburg University (n=1). The departments or institutes from which no survey responses were obtained were contacted again by mail or telephone to guarantee the participation of every institution. A database was established using the information provided and subsequently evaluated. With respect to data evaluation, a SWOT analysis [24] considering strength and weaknesses (internal perspective) as well as opportunities and threats (external perspective) for the strategy of establishing a medical education research network at the UKE was performed by the authors as an additional analysis tool to generate recommendations based on this analysis.

## Results

Thirty-two of the 65 institutions replied to the online survey. All remaining institutions were contacted by email or telephone until a response from every institute and department had been gathered. Most of the institutions who did not reply to the online survey initially were not involved in educational projects. 

**Figure 1:**
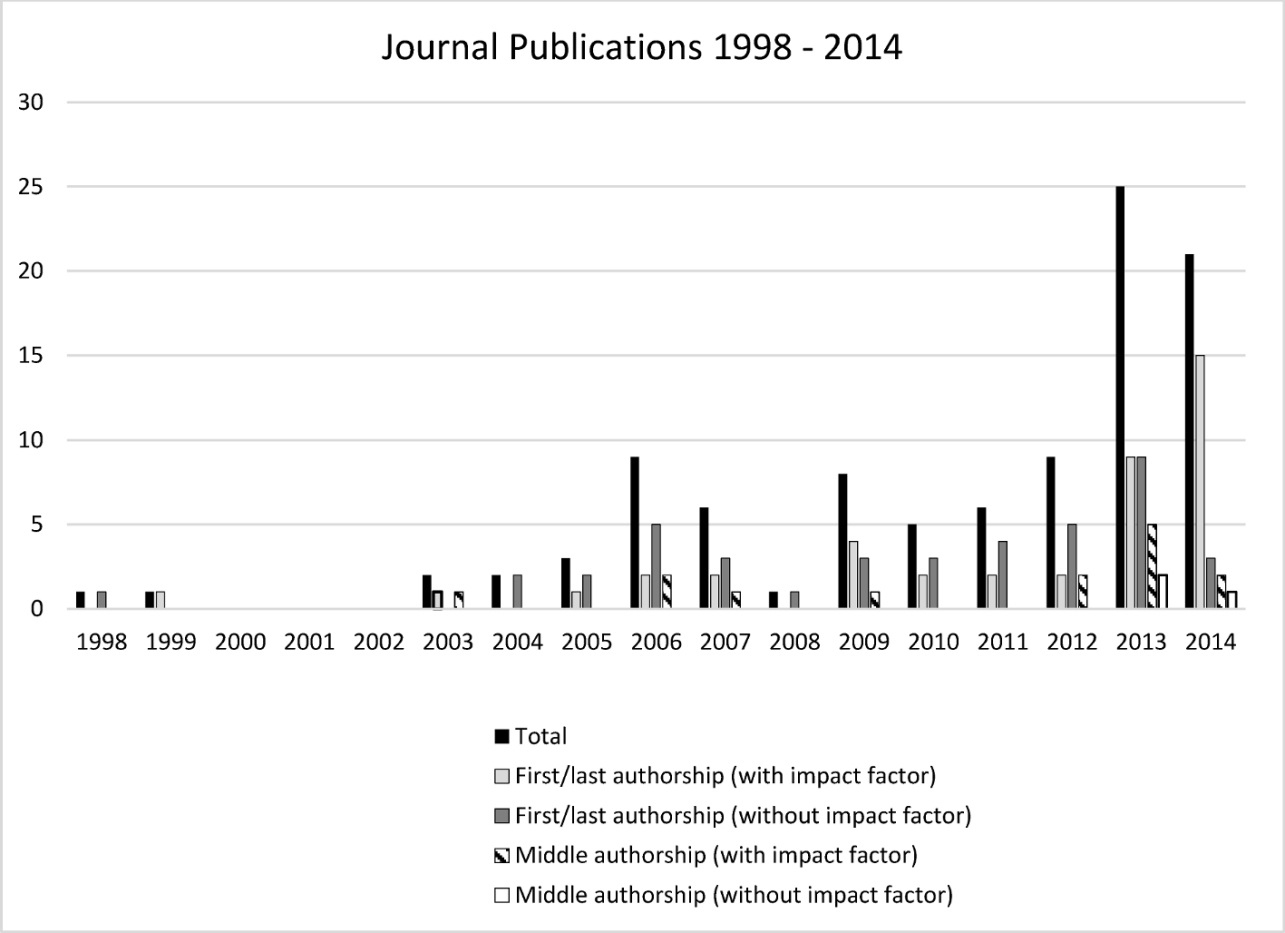
Medical education publications at the Medical Faculty of Hamburg University from 1998 to 2014

Sixty medical education researchers who had been involved in 112 publications in the field of medical education from 1998 to 2014 – 25 of them as first or last authors – were identified in 23 departments or institutes at the Medical Faculty of Hamburg University. Among these manuscripts, 99 (88.4%) were published in journals (see Figure 1 (Fig. 1)) and 13 (11.6%) were contributions to books. The first publication in medical education at the UKE dated back to the year 1998. From 2000 to 2002, no manuscripts on medical education were published. The individual number of journal publications per person varied from one to 43.

At the time of the survey, 24.6% of the departments and institutes at the Medical Faculty of Hamburg University were involved in educational research projects and 46.2% participated in educational service projects. Research projects were defined as projects with a scientific question and a description of methodical proceeding. Service projects were defined as projects that implement new courses or educational material without a specific research question. In total, at least 42 ongoing research projects and 57 service projects were identified. Table 1 (Tab. 1) shows the different topics of the current medical education research projects. The target groups mentioned in this multiple-answer question are mostly undergraduate medical students in their preclinical (42.4%) and clinical (53.1%) years or in their practice year (31.2%). Individual research projects address physicians in postgraduate training, pupils, university applicants, psychotherapists or nursing students. In another multiple-answer question, 46.9% of the participants used quantitative research methods and 45.5% used qualitative methods to answer their research questions. Mixed-method approaches were only employed by 17.6% of the participants. 

Currently, 33 institutions are involved in medical education projects at the UKE. Four institutions receive funding for educational research projects, 18 for educational service projects and three for projects of both types. Hence, 75.8% of the institutions have received funding for their educational projects (education fund of the Medical Faculty: 96%, Federal Ministry of Education and Research and Ministry of Education and Science: 12%, Foundations: 8%). Eight institutions have received no funding for their medical education projects. 

Between 2005 and 2014, 14 medical or dental and one non-medical doctoral theses with research questions in the field of medical education were completed. Nineteen doctoral theses (four of them in a PhD program) are currently in progress, including medical, dental and non-medical doctoral candidates. One postdoctoral lecture qualification in the field of medical education was completed in 2014. Ten current faculty members graduated in a Master of Medical Education (MME) program (five in Heidelberg, five in Bern), and six are currently studying in Heidelberg. Seven MME participants (five with MME-diploma and two without) have left the UKE. One faculty member holds a professorship for internal medicine/educational research, and three faculty members hold professorships with preponderant teaching obligations.

The survey participants were also asked to express their expectations, needs and wishes with respect to a possible organizational structure for medical education research at the Medical Faculty of Hamburg University (see Table 2 (Tab. 2)). Regular meetings regarding “work in progress”, finding partners for cooperation, and workshops on educational research methods were the most frequently mentioned topics.

**Figure 2:**
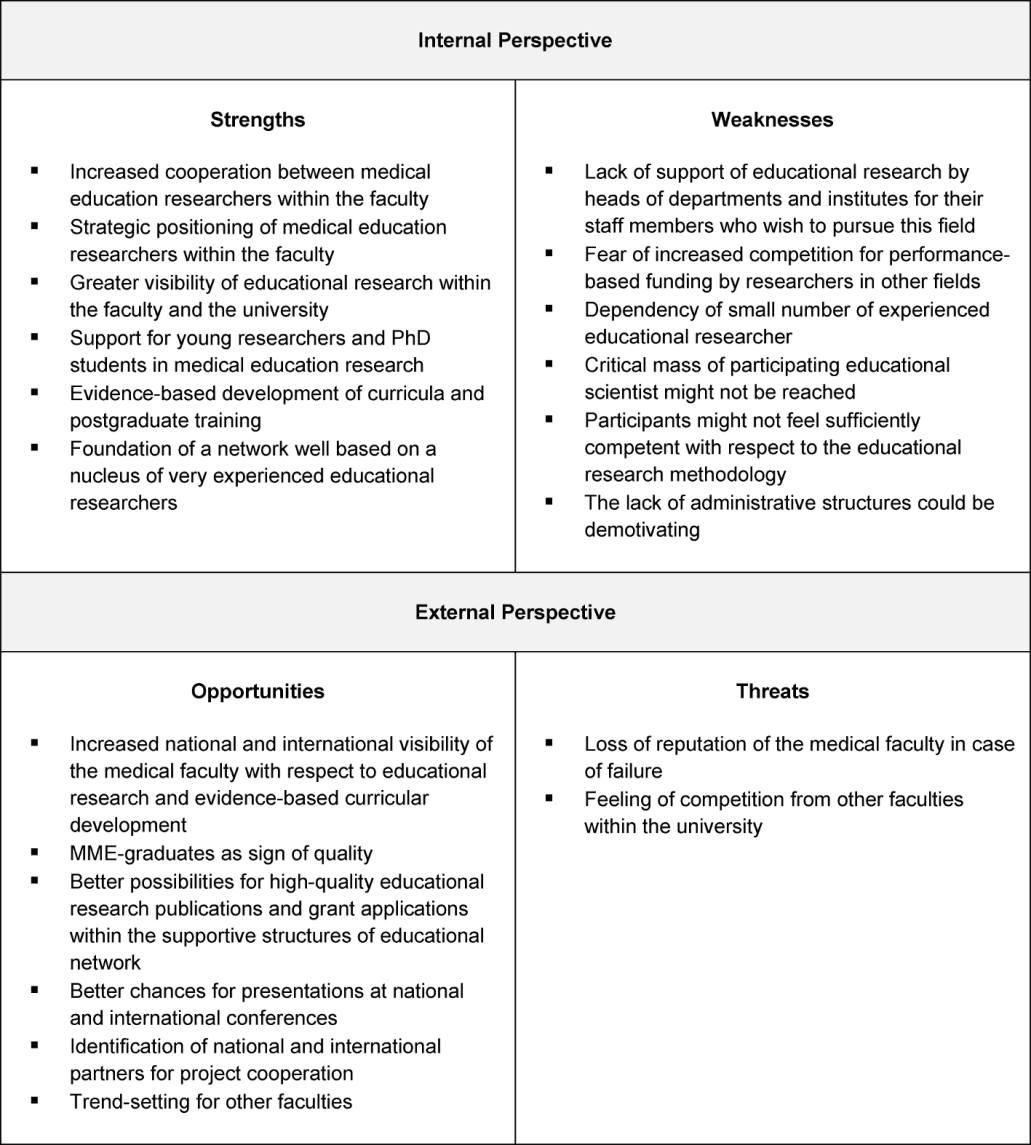
SWOT analysis for establishing a faculty network for medical education research

Based on these data, a SWOT analysis was performed by the authors with respect to the strengths and weaknesses (internal perspective) as well as opportunities and threats (external perspective) related to the possible establishment of a network of educational research at the Medical Faculty of Hamburg University (see Figure 2 (Fig. 2)).

## Discussion

With its current output of publications in the field of medical education, the Medical Faculty of Hamburg University is among the five German universities with more than 10 first or last author publications in international education journals from 2004 to 2013 [20]. This rate of publications seems to be a good foundation for international visibility and a unique selling point that is currently not shared by many other universities in Germany. Furthermore, the increasing number of MME graduates, the number of 42 current educational research projects, and increased third-party funding for educational projects underscore the faculty’s striving for research based medical education. Having identified 25 medical education researchers at the UKE who published at least one manuscript with an educational topic as first or last author, the formation of an educational network appears to be a helpful next step to support the research activities and publications of the these authors, according to our SWOT analysis. However, only three of the 25 researchers published more than 10 manuscripts in national or international journals as first or last authors. This raises the question whether a critical mass of experts, which is crucial for developmental undertakings [25], can currently be achieved at the UKE for such an enterprise. We identified the dependency on a small number of experienced educational researcher as a potential weakness. However, a network with a supportive infrastructure – the lack of which has also been identified as a weakness – might support and motivate young education researchers who are just starting to publish or who hold MME degrees to pursue education research.

In the mid-1990s, the results of medical educational research were often neglected in educational decision-making [26]. Hence, many changes that lack the support of research evidence have been implemented in medical curricula. Thus, the idea that medical education departments should “promote and sustain medical education research” [26] was generated to support curriculum developers with educational research data to provide best evidence medical education [27]. Recommendations have been provided regarding how to establish departments of medical education and support educational researchers [13]. Some of the recommendations for setting up departments of medical education and for evidence-based progress in medical education [28] also address the following supportive necessities to tackle weaknesses that were identified in our SWOT analysis for setting up an education research network. Firstly, support from the dean is required for such an enterprise because faculty member resistance might be expected with respect to a lack of support for researchers from their department with an interest in educational research. Secondly, financial support may be necessary to establish an administrative infrastructure for a network. Thirdly, a multi-professional team of researchers must be willing to share their expertise to educate young academics. 

When the process of “unfreezing” [29] of the faculty is successfully underway and the decision is made to establish a network, several additional recommendations concerning how to successfully conduct collaborative research in medical education [30] must be considered. The fear of increased competition between researchers, as addressed in our SWOT analysis, could be met with the early development of authorship criteria [30]. If financial support is not immediately available, it might be necessary to identify enthusiastic faculty members who will work without funding during the start-up period [30]. Furthermore, like for basic science research, it will be necessary to receive funding for educational research from external sources, which requires expertise in educational grant proposal writing [31] and the identification of funding sources. Regarding the distribution of educational research findings and ensuring their availability for the development of curricula, research in the USA has demonstrated the utility of various national organizations and the Society of Directors of Research in Medical Education [32]. Such an approach will be especially necessary in Germany with respect to the integration of the NKLM into undergraduate medical education and the development or pursuit of model curricula.

An analysis similar to ours to establish the status quo regarding medical education research at a medical faculty could be the first step for a faculty to decide whether it would like to proceed in creating a network or other structure for improved modes of operation. A faculty needs to be aware that research in medical education seeks to deepen our understanding of learning and is not only interested in solving concrete local problems [33]. It can provide evidence that should be considered during the development of an undergraduate medical curriculum [34]. However, setting up a strategy and a structure for development of a nucleus and network in medical education research will require the complete commitment and support of the faculty leaders and a critical mass of faculty members who are willing to tackle grant writing, the establishment of projects and the preparation of manuscripts.

## Conclusion

If a medical faculty wishes to consider the establishment of an educational research focus in its portfolio, an analysis of the status quo with respect to medical education research and researchers appears to be an important first step. Once the analysis data are available, a possible next step towards a network for educational researchers could be a workshop comprising faculty members who are interested in educational research. Such a workshop might provide the dean with additional information for the SWOT analysis to contemplate whether the faculty is ready for the full enterprise of educational research or whether smaller additional steps need to be taken first to reach a critical mass of researchers and sufficient support by leading faculty members.

## Competing interests

The authors declare that they have no competing interests.

## Supplementary Material

Online survey to elicit the status quo in the field of medical education research at the Medical Faculty of Hamburg University

## Figures and Tables

**Table 1:**
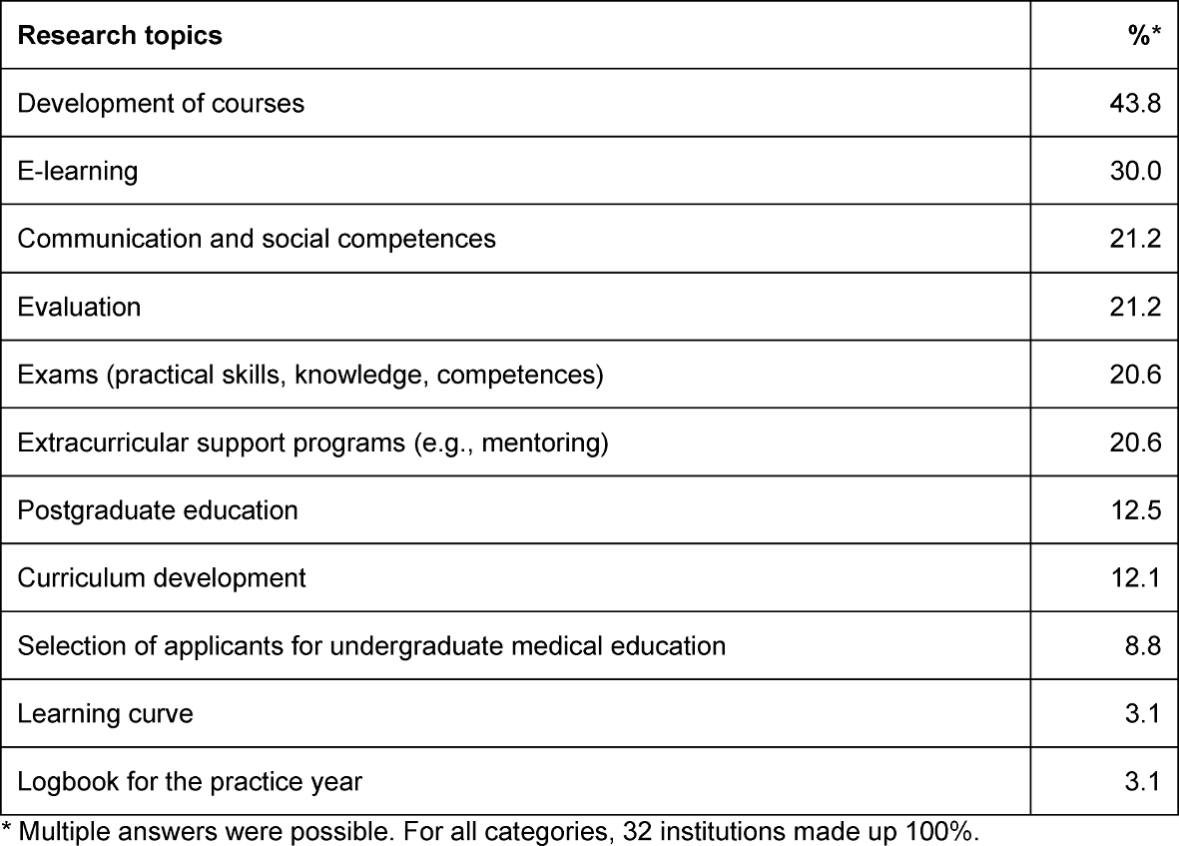
Topics of current medical education research projects

**Table 2:**
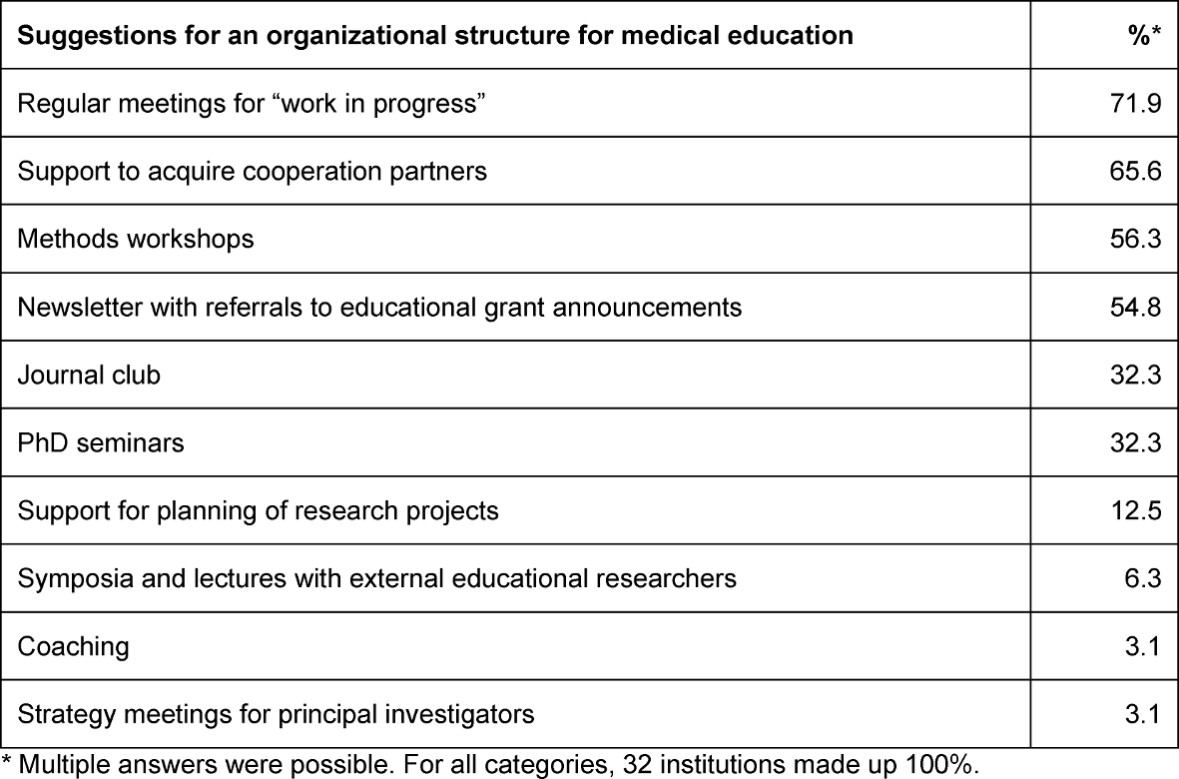
Expectations for an organizational structure for medical education
