# Supplementary material for: Analysis of educational research at a medical faculty in Germany and suggestions for strategic development – a case study
Source: GMS J Med Educ. 2016 Nov 15;33(5):Doc71. doi: 10.3205/zma001070 (PMC5135420; doi:10.3205/zma001070)
Supplement: Online survey to elicit the status quo in the field of medical education research at the Medical Faculty of Hamburg University [file JME-33-71-s-001.pdf]

Online survey to elicit the status quo in the field of medical education research at the Medical Faculty of Hamburg University

Information about the institution

1 Center:

2 Institute/Department:

Projects in the field medical education research at your institute/department

3 Are there current research projects in medical education with the aim of publication at your institute/department?

- ☐ Yes
- ☐ No

4 If yes, how many?

5 Are there current projects to introduce new teaching approaches in medical education? (Without the aim of scientific publication)

- ☐ Yes
- ☐ No

6 If yes, how many?

7 Which persons are currently actively involved in research at your institute/department about topics in the field of medical education? (Please list)

8 Do you receive financial support for medical education by third-party funds at your institute/department? (Multiple answers possible)

- ☐ No
- ☐ Yes, by the German Research Foundation (DFG)
- ☐ Yes, by the German Federal Ministry of Education and Research (BMBF)
- ☐ Yes, by the Teaching Fund of the Medical Faculty (Förderfonds Lehre)
- ☐ Yes, by others

9 If you receive other financial support for medical education, by whom?

Online survey to elicit the status quo in the field of medical education research at the Medical Faculty of Hamburg University

10 Do cooperations exist in the field of medical education? (Multiple answers possible)

- ☐ With groups/persons within the UKE
- ☐ With the University of Hamburg
- ☐ With institutions in Germany
- ☐ With institutions from European countries
- ☐ International
- ☐ There are no cooperations.

11 If cooperations in the field of medical education exist within the UKE, with whom are they?

Topics/research questions in medical education research

12 Which topics in the field of medical education are currently researched at your department?
(Multiple answers possible)

- ☐ E-learning/new media
- ☐ Development of courses
- ☐ Evaluation
- ☐ Extracurricular support programs
- ☐ Communication and social competences
- ☐ Curriculum development
- ☐ Exams: practical skills/competences
- ☐ Exams: theoretical competences/knowledge
- ☐ Selection of applicants for undergraduate medical education

13 Which other topics/research questions in the field of medical education have been/are researched?

14 Which target groups are investigated? (Multiple answers possible)

- ☐ Pupils
- ☐ Applicants for undergraduate medical education
- ☐ Students (preclinical semesters)
- ☐ Students (clinical semesters)
- ☐ Students in the Practice Year
- ☐ Physicians in postgraduate training

15 Other target groups?

16 Please list all published articles and book contributions in the field of medical education with participation of members from your institute/department.

Applied methods

17 Which research methods are/were applied? (Multiple answers possible)

- ☐ Quantitative analytical methods
- ☐ Qualitative analytical methods
- ☐ Mixed methods

18 Additional information about applied methods/research design (e.g. cross-sectional or longitudinal research design, content analysis, comparison of means, regression analysis, ...)

MD thesis/PhD studies in the field medical education research

19 Are/were there MD theses or PhD studies in the field of medical education research at your institute/department? (Multiple answers possible)

- ☐ Yes, publication-based doctorates
- ☐ Yes, monograph-based doctorates
- ☐ No, no doctorate studies are/were conducted in this field.

20 If yes, please list all titles of all finished and current doctorate studies.

Online survey to elicit the status quo in the field of medical education research at the Medical Faculty of Hamburg University

Center of Medical Education Research

21 What would be your wishes for a newly founded Center of Medical Education Research?

22 Which offers would you desire? (Multiple answers possible)

- ☐ Meetings for exchange about "work in progress"
- ☐ Journal Club
- ☐ Methods workshops
- ☐ Doctoral/PhD-Colloquium
- ☐ Support with finding cooperation partners
- ☐ Newsletter to national and international funding opportunities

23 Which other offers would be interesting for you?

23 Do you have other suggestions or wishes with respect to medical education research at the UKE?
